# Shape-Changing Tubular Hydrogels

**DOI:** 10.3390/gels4010018

**Published:** 2018-02-22

**Authors:** Srinivasa R. Raghavan, Neville J. Fernandes, Bani H. Cipriano

**Affiliations:** Department of Chemical & Biomolecular Engineering, University of Maryland, College Park, MD 20742, USA; nevillefernandes87@gmail.com (N.J.F.); bhcipriano@gmail.com (B.H.C.)

**Keywords:** hybrid hydrogels, stimuli-responsive polymers, smart materials

## Abstract

We describe the creation of hollow tubular hydrogels in which different zones along the length of the tube are composed of different gels. Our method to create these gels is adapted from a technique developed previously in our lab for creating solid hybrid hydrogels. The zones of our tubular gel are covalently bonded at the interfaces; as a result, these interfaces are highly robust. Consequently, the tube can be picked up, manipulated and stretched without suffering any damage. The hollow nature of these gels allows them to respond 2–30-fold faster to external stimuli compared to a solid gel of identical composition. We study the case where one zone of the hybrid tube is responsive to pH (due to the incorporation of an ionic monomer) while the other zones are not. Initially, the entire tube has the same diameter, but when pH is changed, the diameter of the pH-responsive zone alone increases (i.e., this zone bulges outward) while the other zones maintain their original diameter. The net result is a drastic change in the shape of the gel, and this can be reversed by reverting the pH to its original value. Similar localized changes in gel shape are shown for two other stimuli: temperature and solvent composition. Our study points the way for researchers to design three-dimensional soft objects that can reversibly change their shape in response to stimuli.

## 1. Introduction

Recently, our lab has been interested in devising new polymeric hydrogels that exhibit different properties over distinct zones [1,2]. For example, we have created gels in which one zone or portion is responsive to a particular stimulus, say temperature, while adjacent zone(s) are unresponsive to this stimulus (and possibly responsive to a different stimulus such as solution pH) [1,2]. In addition, we have designed gels in which adjacent zones have very different mechanical properties: e.g., a stiff, brittle zone can be next to a soft, extensible zone [1]. The motivation for these studies arose from our realization that many gel-like systems in nature, including both aquatic and terrestrial animals, tissues or organs in our body, and plant parts, are inhomogeneous and have multiple zones with distinct properties [3,4]. Moreover, we noted that current approaches to create novel hydrogels were mainly focused on tuning the chemical structure of the monomers used in gel synthesis [5,6]. In comparison, physical approaches to creating hybrid hydrogels have been relatively unexplored [7,8,9,10], and we have therefore focused our efforts in this direction.

Another direction in the hydrogel field that has proven popular among researchers is in designing hydrogels that change their shape in response to a stimulus [11,12,13]. The natural inspiration for shape-changing materials comes from a variety of plant-based structures such as seed pods, awns, and leaves, all of which exhibit changes in shape under specific conditions. Most studies on shape-changing gels begin with a flat film or strip of a gel, which folds, i.e., rolls up, to form a particular shape, most typically an open tube [11,12,13]. Folding has been induced in response to external stimuli such as temperature [14], pH [15], or light [16], or upon the addition of aqueous solutes such as enzymes [17]. Gels that fold usually have a hybrid design, where the flat sheet is itself composed of multiple zones or layers. This is done by cross-linking monomers using ultraviolet (UV) light that is sent through a photolithographic mask corresponding to a specific pattern. The ability of a gel to fold hinges on the differential swelling of various zones in the gel when placed in water; this in turn creates stresses in the material that lead to folding. To our knowledge, gels that fold are always a flat sheet at the outset, *not* a three-dimensional (3-D) object. 

In this paper, we report the creation of hybrid polymer gels in a tubular geometry. Unlike the solid hybrids reported earlier from our lab (which were either cylinders, discs, or cuboids) [1,2], the hybrids in this study are cylindrical *tubes*–with a hollow core and a wall that is thin compared to the cylinder diameter. Along its length, each tube has at least two zones, which correspond to different polymer gels. The properties of the overall tube thus depend on the chemistry of each zone. We will discuss the responses of specific tubes to three external stimuli: temperature, pH, and solvent composition. The main result here is that our tubes exhibit shape changes, which is due to the differences in swelling between the distinct zones of the tube. Thereby, this study, for the first time demonstrates *reversible changes from one 3-D shape to another in a polymer gel*. Additionally, our study shows that hollow cylinders exhibit a change in their volume and shape much faster than solid ones, which is a crucial benefit of working with the former.

## 2. Results and Discussion

### 2.1. Synthesis of Hybrid Gel Tubes

**Figure 1:**
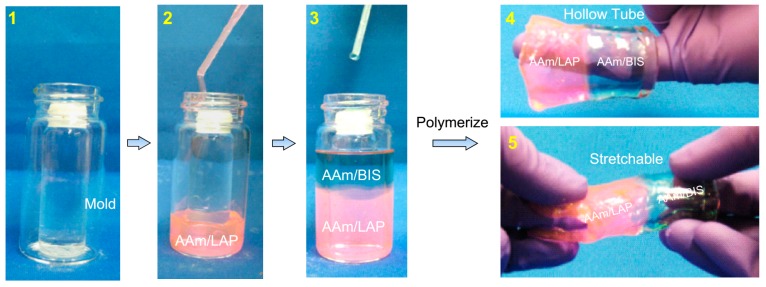
Procedure for preparing hybrid gel tubes with distinct zones. (**1**) A mold is created by placing or gluing a smaller glass vial within a bigger one. (**2**) A highly viscous pre-gel solution of acrylamide (AAm)-laponite (LAP) colored with a pink dye is pipetted into the annular space in the mold. (**3**) The second pre-gel solution of AAm-*N,N*′-methylene-bis(acrylamide) (BIS) is then gently pipetted over the first pre-gel so as not to induce convective mixing at the interface. The system is then left to polymerize at room temperature. Thereafter, the gel is extracted from the mold. (**4**) The gel is shown to be a hollow tube with two distinct zones: AAm-LAP (pink) and AAm-BIS (colorless). (**5**) The gel can be gripped using one’s fingers, and can be stretched without breaking. The AAm-LAP zone is more extensible than the AAm-BIS zone.

Our method for preparing hybrid tubular gels is illustrated in Figure 1. This method was developed previously in our lab [1] and has been subsequently copied and extended by others [9,10]. The key to this method is to stack the pre-gel solutions when their viscosities are sufficiently high, so as to drastically minimize convective mixing between the solutions [1]. To prepare a tubular hybrid, we use a mold so that the center of the gel remains hollow. The simplest version of the mold is made by arranging a small cylinder (vial or tube) concentrically inside a bigger cylinder, as shown in Panel 1. The small vial here is filled with water to increase its inertia and sealed with tape. The pre-gel solutions are then pipetted in the annular space between the two vials, one on top of the other. We first introduce pre-gel A, a mixture of monomer, cross-linker, initiator, accelerant, and rhodamine B dye up to half the vial height (Panel 2). Here, the monomer is acrylamide (AAm) at a concentration of 1 M (~7% by wt) and the cross-linkers are laponite (LAP) nanoparticles (4 wt %). Pre-gel A is already a viscous solution at the outset. Next, pre gel B, a solution of 1 M AAm and 0.1 wt % *N,N′*-methylene-bis(acrylamide) (BIS) along with the same initiator and accelerant, is introduced on top of pre-gel A (Panel 3). Note that the two pre-gels in this case have the same monomer, but different cross-linkers; also, pre-gel B is colorless whereas pre-gel A has a pink color from the dye. The high viscosity of pre-gel A prevents convective mixing at the interface between the pre-gels [1].

We then leave the system to polymerize at room temperature. Afterward, the gel is removed from the mold and is shown to be a hollow tube with a diameter of ~25 mm, length of ~50 mm and a wall thickness of ~ 5 mm (Panel 4). Note that the inner vial diameter dictates the wall thickness and the outer vial diameter dictates the outer diameter of the tube. The tube shows well-separated regions of the two gels, i.e., AAm/LAP is the pink zone and AAm/BIS is the colorless zone. As expected, the zone that is cross-linked by LAP is more compliant and stretchable than the adjacent zone that is cross-linked by BIS (Panel 5) [1,18]. Note that the LAP serves as chemical cross-linkers here: i.e., when AAm monomers and LAP particles are in the presence of free-radicals generated by the initiator, polymer chains of AAm are induced to grow from the surfaces of LAP particles [18,19]. The higher stretchability of LAP gels is believed to be because the chain segments between adjacent particles (i.e., cross-links) are longer than in a conventional BIS cross-linked gel [1,16]. 

It is important to note that the zones of the hybrid tube are connected by a strong and robust interface. As a result, the tube does not rupture on twisting or stretching (Panel 5). The robust interface is a consequence of our synthesis method [1]. When viscous pre-gels A and B are brought into contact (Panel 3), chains (oligomers) of A will be able to diffuse from Zone A into Zone B and vice versa. As a result, some covalent linkages of A and B chains will occur at the interface, which are crucial in ensuring that the interface is robust [1]. If the pre-gels are not viscous, they will undergo considerable mixing and one will end up with a gel that is a copolymer of A and B, rather than well-separated zones. Also, if we fully polymerize Gel A and Gel B and thereafter bring them into contact, the A/B interface will be very weak and the sample will be ripped apart under moderate stretching [1].

### 2.2. Swelling Kinetics for Solid Cylinders vs. Hollow Tubes

**Figure 2:**
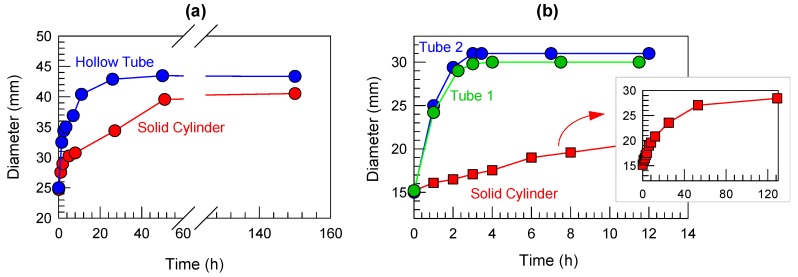
Comparing the swelling kinetics of solid cylinders and hollow tubes of identical composition. All structures are prepared using AAm:2-(dimethyl-amino)ethyl methacrylate (DMEM) 90:10 and are cross-linked with BIS. The DMEM is a cationic monomer that imparts ionic character to the gels. At *t* = 0, the structures are placed in water at pH 7, whereupon they swell, and their outer diameters are measured as a function of time. (**a**) Initial diameter of the cylinder and tube are 25 mm. The tube has a wall thickness of 5 mm. (**b**) Initial diameter of the cylinder and tubes are 15 mm. Tubes 1 and 2 have wall thicknesses of 1.8 and 1.2 mm, respectively. The solid cylinder takes a much longer time to reach an equilibrium size and the full data set for this sample is plotted in the inset.

How does the rate of swelling compare between solid and hollow cylinders? To study this, we made gels in the form of solid cylinders and hollow tubes with the same monomer composition (both gels were not hybrids). The monomer was a mixture of AAm (nonionic) and 2-(dimethylamino)ethyl methacrylate (DMEM) (cationic), with the AAm:DMEM molar ratio fixed at 90:10. The total monomer content was 1 M and the cross-linker was 0.1% BIS. Due to its ionic nature, a gel with this composition is expected to swell significantly in water at pH 7 [5,6]. Figure 2 shows two comparisons of solid and hollow cylinders, which were each placed in a reservoir of water at time *t* = 0. In Figure 2a, the two cylinders have an outer diameter of 25 mm and a length of 50 mm, with the wall thickness of the hollow tube being 5 mm. The solid cylinder swells to a diameter of 40.5 mm in about 150 h. The hollow tube swells to a slightly larger diameter of 43.4 mm, but more importantly in only about 50 h, i.e., in one-third the time. In Figure 2b, the three cylinders all have an outer diameter of 15 mm and a length of 40 mm. Two hollow tubes are studied, with wall thicknesses of 1.8 and 1.2 mm, respectively. The two tubes swell to a diameter of ~30 mm within about 4 h. The solid cylinder, on the other hand, swells to a slightly lower diameter of 28.4 mm, but takes more than 120 h to do so (30-fold longer).

The above data clearly show the faster swelling of hollow tubes compared to their solid cylinder counterparts. This is due to the fact that the smallest dimension pertinent to swelling of the hollow gels is the wall thickness, which is 1 to 5 mm. The relevant counterpart for the solid gels is the outer diameter of the cylinder, which is either 15 or 25 mm, i.e., a much larger dimension. Diffusion in the main mode by which water is transported into the gel, allowing it to swell. It is well-known that the diffusive timescale *τ* will vary with the smallest dimension *a* of the gel as per the Einstein-Smoluchowski equation [20]:

(1) $$\tau \;\sim \;\frac{a^{2}}{Ɗ}$$

where Ɗ is the diffusivity of the species that is diffusing, which in this case is water. Equation (1) shows that the smaller the length *a* to be diffused through, the lower the time *τ* for diffusion.

### 2.3. Stimuli-Responsive Gel Tubes (Two-Zone Hybrids)

**Figure 3:**
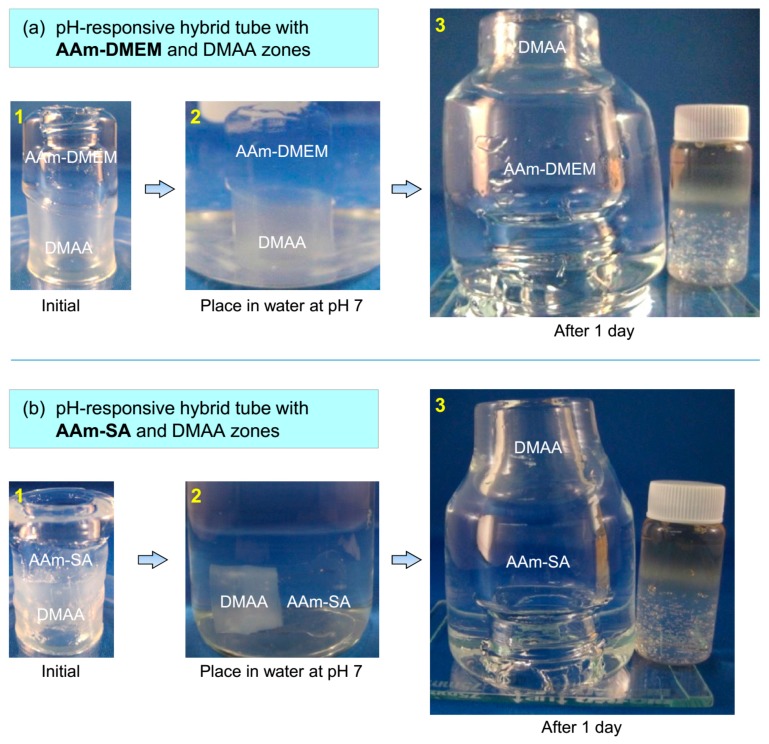
Shape changes in hybrid gel tubes induced by pH. The tubes in (**a**,**b**) have two zones, of which one is pH-responsive. (**a**) The pH-responsive zone is made from AAm:DMEM 90:10 (DMEM is cationic). (**b**) The pH-responsive zone is made from AAm:SA 90:10 (SA is anionic). In both cases, the second zone of the tube is made from DMAA (nonionic). (**1**) The initial as-made tube. (**2**) Tube immersed in water at pH 7. (**3**) After more than a day, the tube is removed from water and photographed next to a vial for size comparison (the vial and the initial tube have similar size). In both cases, the ionic zone is seen to have swollen a lot more than the nonionic zone, and the overall tube thus assumes a bottle-like shape.

We proceeded to investigate hollow hybrid tubes having two zones with different stimuli-responsive properties. First, we created tubes with one ionic and one nonionic zone and studied the effect of pH on these tubes. Two such tubes are shown in Figure 3. The nonionic zone in each tube is made using 1 M of *N*,*N*′-dimethylacrylamide (DMAA) with 7.5% LAP as the cross-linker. For the tube in Figure 3a, the ionic zone is composed of AAm:DMEM in a molar ratio of 90:10 (the total monomer being 1 M) and with 0.1% BIS as the cross-linker. As noted in Figure 2, DMEM is a cationic monomer and imparts ionic properties to its zone. The reason for the high LAP content in the nonionic DMAA zone was to inhibit its swelling. Figure 3a, panel 1 shows the initial hybrid tube, which has dimensions identical to the tube in Figure 1, i.e., 25 mm outer diameter, 5 mm wall thickness, and overall height 50 mm. The nonionic and ionic zones are each about half the height of the tube, i.e., 25 mm each. 

The above tube is then placed in water at ambient pH and temperature (Panel 2), and it is left to swell for more than a day. Thereafter, the swollen tube is removed and placed vertically next to a vial for size comparison (the vial is 25 mm × 55 mm, i.e., the size of the initial tube) (Panel 3). We observe substantial swelling in the ionic zone of the tube compared to the nonionic zone. The ionic zone has increased to about 4× its original diameter and 2× its original height whereas the nonionic zone is only slightly larger than its original dimensions. The overall gel thus assumes the shape of a bottle with a small neck relative to its body. Similar results are obtained for a different hybrid tube where we use the anionic monomer sodium acrylate (SA) instead of the cationic DMEM (Figure 3b). This tube has the same DMAA/LAP zone and a zone of 1 M AAm:SA in a 90:10 (1 M total monomer) cross-linked with 0.1% BIS. When this tube is placed in water at ambient pH, it also shows substantial swelling of its ionic zone relative to its nonionc zone (Panel 3). Thus, the tube again transforms to a bottle shape, and here the diameter of the ionic zone reaches about 3× its original diameter. In both the above cases, the original dimensions of the tube can be recovered by altering pH. For the tube in Figure 3a, it has to be placed in water at pH 10 or greater, whereupon the DMEM-bearing chains lose their charge. The ionic zone then deswells and reverts to its initial size. For the tube in Figure 3b, similar deswelling occurs when it is placed in water at pH 3 or lower, whereupon the SA units lose their charge.

**Figure 4:**
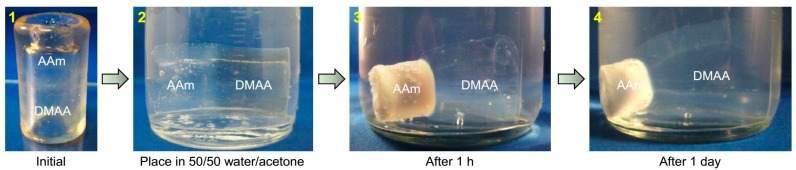
Shape changes in hybrid gel tube induced by solvent composition. (**1**) The initial as-made tube has two zones: one of DMAA and the other of AAm. The AAm zone alone is sensitive to solvent composition. (**2**) The tube is immersed in a 50/50 water/acetone mixture at *t* = 0. (**3**) Within 1 h, the AAm zone is completely opaque and begins to shrink. (**4**) After 1 day, the opaque AAm zone has shrunk to half its original size, whereas the DMAA zone has swelled up. The overall tube has a funnel shape due to the different diameters of the two zones.

Next, we studied a hybrid tube that is responsive to solvent composition (Figure 4). In this case, the hybrid has two zones, one of 1 M AAm cross-linked with 3% LAP and another of 1 M DMAA cross-linked with 4% LAP (Panel 1). It is known that AAm gels shrink in water containing acetone above a critical level (~50%) whereas DMAA gels are not sensitive to acetone [5,6]. This behavior derives from the fact that poly(AAm) chains are soluble in water but insoluble in acetone. We placed the AAm/DMAA hybrid tube in a 50/50 acetone/water solution at room temperature (Panel 2). As shown by Panels 3 and 4, the AAm zone shrinks and turns opaque, while the DMAA zone slowly starts swelling to absorb the solvent. After a day, the AAm zone is considerably smaller than its initial size while the DMAA portion is swollen appreciably. Thus, the initial tube is transformed into a funnel shape with a significant difference in diameter between the two zones. This shape change can be reversed by placing the tube back in water.

**Figure 5:**
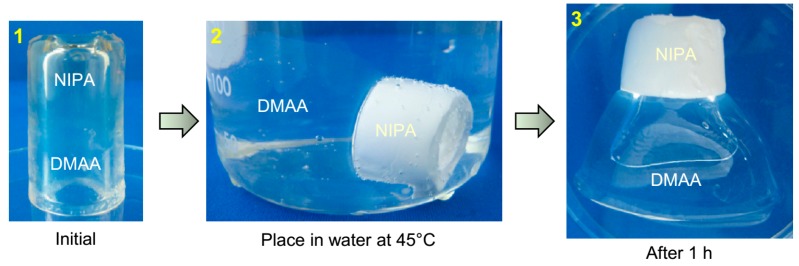
Shape changes in hybrid gel tube induced by heating. (**1**) The initial as-made tube has two zones: one of *N,N*′-dimethyl-acrylamide (DMAA) and the other of *N*-isopropylacrylamide (NIPA). The NIPA zone alone is sensitive to temperature. (**2**) The tube is immersed in hot water (45 °C) at *t* = 0. Immediately, the NIPA zone of the gel starts shrinking and turns opaque whereas the DMAA zone remains clear. (**3**) After 1 h, the opaque NIPA zone has shrunk appreciably whereas the DMAA zone has swollen and expanded a bit. The overall tube has a funnel shape due to the different diameters of the two zones.

We then explored a temperature-responsive hybrid tube (Figure 5). This again had two zones, one of 1 M *N*-isopropylacrylamide (NIPA) cross-linked with LAP (3%) and another of 1 M DMAA cross-linked with LAP (4%) (Panel 1). NIPA gels are known to be thermo-responsive: specifically, they shrink when heated above their lower-critical solution temperature (LCST), which is 32 °C [5,6]. DMAA gels, in contrast, are not responsive to temperature. Panels 2 and 3 show the result of placing the NIPA/DMAA tube in hot water (~45 °C), above the LCST of NIPA. The NIPA zone turns opaque and shrinks, whereas the DMAA zone remains clear and expands slightly. Thus, the tube is again transformed into a funnel shape. On placing the gel back in water at room temperature (23 °C, below the LCST of NIPA), the NIPA portion reverts to its initial clear state and the gel recovers its symmetric tubular shape.

**Figure 6:**
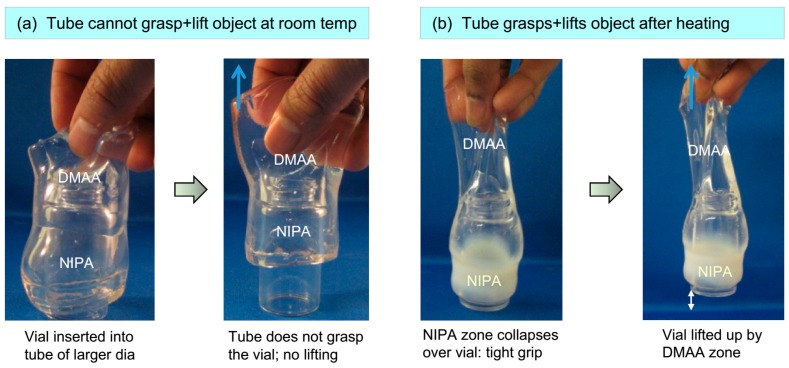
Grasping an object using a shape-changing gel tube. The tube has two zones: DMAA and NIPA, of which only the latter is responsive to temperature. (**a**) A vial of slightly smaller diameter is inserted into the tube. The tube does not tightly grasp the vial; therefore, when the tube is lifted up, it slides over and does not pull up the vial. (**b**) When the vial/tube combination is immersed in hot (50 °C) water, the NIPA zone of the tube contracts and collapses around the vial, thereby exerting a tight grip. When the tube is lifted up by its free DMAA portion, the vial can now be picked up along with the tube. The photo on the right shows that the vial has been lifted off the surface.

The shape-changing properties of the above tubular gels can be potentially exploited for certain applications. We illustrate one such idea in Figure 6. For this, we created a hybrid tube with one zone of DMAA cross-linked with 7.5% LAP and the other of NIPA cross-linked with 3% LAP. The inner diameter of the tube was designed to be slightly more than 15 mm. A vial of 15 mm outer diameter was then inserted through the center of the tube. In this state, the tube is not able to grasp the vial (Figure 6a). But when the tube-vial combination is immersed in hot water at 50 °C, the shrinking of the NIPA zone allows this portion to contract around the vial, grasping it tightly. This happens within minutes after immersion. The DMAA zone remains free and unadhered to the vial. When the tube is now pulled up by its DMAA zone, it is able to lift the grasped vial off the ground cleanly and without any slippage (Figure 6b). Note that the DMAA portion is stiffer and less elastic due to the higher cross-linker content, which is ideal for the lifting zone. The key result here is that the responsive tube is able to alter its shape to conform to the shape of the encapsulated object. As a result, the tube is able to grasp the object tightly, allowing it to be picked up and manipulated. This ability could be potentially useful in soft robotics or related areas.

### 2.4. Stimuli-Responsive Gel Tubes (Three-Zone Hybrids)

**Figure 7:**
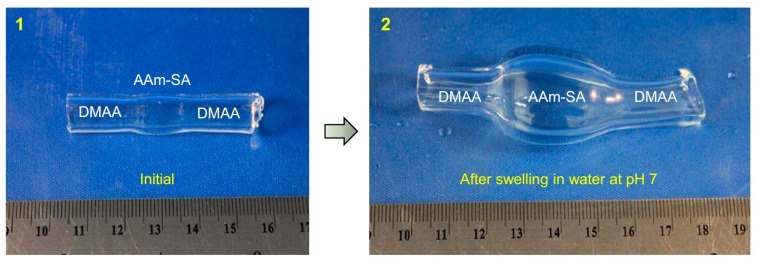
Shape changes in a three-zone hybrid gel tube induced by pH. (**1**) The initial as-made tube has three zones: a central zone of AAm:SA 90:10 that is pH-responsive due to the anionic SA, and two zones flanking this that are made from the nonionic DMAA. The diameter is uniform across the three zones. (**2**) After swelling in water at pH 7 for more than a day, the tube is removed and photographed. A ruler is shown in both photos for size comparison. The photo shows that the middle ionic zone is significantly expanded in both diameter and length compared to its initial size and compared to the adjacent nonionic zones. Thus, there is a distinct change in the shape of the hybrid tube.

The procedure outlined here to create hybrid tubular gels can be extended in many ways. One extension that we will now discuss is to create a tube with more than two zones. This is shown by the tube in Figure 7, which has three adjacent zones, of which only one is pH-responsive. The zone compositions are as in Figure 3b: the left and right zones are each made of 1 M DMAA cross-linked with 7.5% LAP, while the middle zone is made of 1 M AAm:SA 90:10 cross-linked with 0.1% BIS. This tube is synthesized by the same procedure as in Figure 1, but modified to allow for three zones instead of two. As synthesized, the overall tube length is 50 mm, with the DMAA zones each having a length of 20 mm each while the AAm:SA zone has a length of 10 mm (Panel 1). 

We then placed this tube in water at pH 7 and left it to swell for more than a day. Thereafter, the swollen gel is removed and placed next to a ruler for size comparison with the initial tube (Panel 2). At a pH of 7, the middle zone (AAm:SA) swells due to the anionic nature of the SA groups in it, but the other two zones are nonionic and hence do not swell as much. Due to this differential swelling, the initial tube is transformed into a shape with a central bulge. The diameter and length of the middle zone (the bulged region) are about 3× their original values at their central point. On the other hand, the diameter and length of the flanking zones are only slightly larger than their initial values. This shape change can also be reversed by placing the gel in pH 3 water. Figure 7 shows that multi-zone hybrid tubes can be used to engineer more complex shape-changes than are possible with two-zone hybrids.

## 3. Conclusions

In this paper, we have showcased a new approach for making hybrid tubular gels that have zones corresponding to different polymers along the length of the tube. Each zone retains its unique individual properties. The zones are connected by a robust interface, with polymer chains across the interface being covalently bonded to each other. The hollow nature of the tubes allows them to swell much faster than their solid counterparts. By choosing stimuli-responsive monomers for distinct zones, we can engineer a tube that shows a significant change in shape when exposed to particular stimuli. The three stimuli that were studied here are pH, temperature, and solvent composition. Shape-changes occur because distinct zones of the tube swell (or shrink) to different extents in response to the stimulus. The shape changes are reversed when the stimulus is restored to its initial value. Such reversible shape-changes have been demonstrated with both two-zone and three-zone hybrids, and this concept can be easily extended to even more complex structures with multiple zones. Overall, our study shows how researchers can build a hydrogel in a particular 3-D shape and have it transform into another 3-D shape in a controlled fashion. 

## 4. Materials and Methods 

### 4.1. Materials

The monomers acrylamide (AAm), *N*,*N*′-dimethyl-acrylamide (DMAA) and *N*-isopropylacrylamide (NIPA) were purchased from TCI America. The cross-linker *N,N*′-methylene-bis(acrylamide) (BIS) and the initiator potassium persulfate (KPS) were purchased from Sigma-Aldrich (St. Louis, MI, USA). The accelerant *N*,*N*,*N*′,*N*′-*tetra*-methylethylenediamine (TEMED) was purchased from J. T. Baker (Phillipsburg, NJ, USA). The ionic monomers sodium acrylate (SA) and 2-(dimethyl-amino)ethyl methacrylate (DMEM) were purchased from Sigma-Aldrich. The inorganic clay laponite XLG (LAP) was obtained from Southern Clay Products (Gonzales, TX, USA). All chemicals were used as purchased without any further purification.

### 4.2. Sample Preparation

All gels were prepared using deionized (DI) water that was degassed by exposure to nitrogen. Monomers were dissolved in water to a total concentration of 1 M. The cross-linkers, either BIS or LAP particles, were then added. BIS was usually added at a concentration of 0.1 wt %. LAP content in the solution varied between 3–7.5 wt % with respect to the total weight of the solvent. In the case of LAP, to prevent particle clumping, it was important to add the particles slowly while the solution was being vigorously stirred on a magnetic stirrer plate. Stirring was continued until the mixture appeared homogeneous and translucent. If the need arose, these mixtures would be sonicated or centrifuged for a short time till any dispersed gas bubbles were removed. The initiator KPS and accelerant TEMED were then added in molar concentrations (with respect to total monomer) of 0.426 mol% and 0.735 mol% respectively. As soon as the initiator and accelerant were added, the polymerization reaction began and was allowed to continue in a nitrogen environment (or oxygen-free environment) at room temperature. Although the BIS-crosslinked gels would polymerize within 10–30 min, all gels were left to polymerize for about 20 h before extracting them from the vials/molds.

### 4.3. Hybrid Gel Preparation

The synthesis of hybrid tubular gels is depicted in Figure 1 and is discussed in the accompanying text. A typical procedure is as follows. First, a 5 mL vial of 16 mm diameter and 50 mm length (with cap unscrewed) was filled with water, sealed off with parafilm, and placed inside a bigger 20 mL vial of 25 mm diameter and 55 mm length. The first pre-gel mixture (e.g., AAm-LAP) was poured carefully in the annulus around the smaller vial, with care taken to ensure that no air bubbles were formed. Then the same was done with the second pre-gel (e.g., AAm-BIS). The system was then allowed to polymerize for 20 h at room temperature. The outer vial was then carefully broken and the hollow hybrid gel was extracted and washed. To synthesize solid hybrids, the above procedure was repeated without the inner 5 mL vial.

### 4.4. Swelling Comparison of Solid and Hollow Gels

For the data shown in Figure 2, the solid and tubular gels were washed thoroughly before being immersed in beakers containing water at pH 7 at *t* = 0. The beakers were periodically replenished with water because the water-levels would go down as the gels swelled. Only the outer diameters of the gels were measured as a parameter to gauge the extent of swelling.
